# Perceptions towards childhood asthma and barriers to its management among patients, caregivers and healthcare providers: a qualitative study from Ethiopia

**DOI:** 10.1186/s12890-022-01984-2

**Published:** 2022-05-08

**Authors:** Eden Kassa, Rahel Argaw Kebede, Bruck Messele Habte

**Affiliations:** 1 College of Health Sciences, Defence University, Bishoftu, Ethiopia; 2 Department of Pharmaceutics and Social Pharmacy, School of Pharmacy, College of Health Sciences, Addis Ababa University, Addis Ababa, Ethiopia; 3 Department of Pediatrics and Child Health, School of Medicine, College of Health Sciences, Addis Ababa University, Addis Ababa, Ethiopia

**Keywords:** Children with asthma, Caregivers, Barriers to management, Qualitative study, Ethiopia

## Abstract

**Background:**

The management of asthma, which is one of the major causes of childhood morbidity and mortality has been affected by non-adherence to recommended treatment regimens with severe consequences. The aim of the present study was therefore to explore the perceptions of the children with asthma, their caregivers and their healthcare providers towards asthma and barriers to long term childhood asthma management in an institutional setting in Addis Ababa, Ethiopia.

**Methods:**

A qualitative descriptive design was followed for the present study that used individual interviews as a data collection method. The study participants were 23 pairs of children with asthma that had treatment follow-ups in two tertiary hospitals and their caregivers and eight healthcare providers who cared for these children. The data was analyzed using thematic analysis approach.

**Results:**

The study findings revealed that the children’s reported adherence to the recommended treatment regimens was low and they along with their caregivers were facing physical, emotional and social burdens related to asthma. Some of the influencing factors affecting childhood asthma management were found to be the low-level implementation of the asthma management guidelines by the healthcare providers, limited awareness about asthma and its management by the children and their caregivers, use of traditional home remedies and religious healing on a complementary and alternative basis and inadequate education received from healthcare professionals. Further identified barriers to the adherence of especially inhaled corticosteroids appear to be the low necessity beliefs towards chronic administration of treatment regimens and concerns related with difficulty of administration, fear of side effects and general negative attitude towards it, in addition to their low availability and affordability.

**Conclusions:**

Low awareness of the biomedical treatment regimens and use of traditional home remedies and religious healing by the children with asthma and their caregivers, the low-level implementation of the asthma management guidelines as well as low access to medications may among other things contribute to the low adherence of the children to their recommended regimens. The findings support the need for implementation of asthma management guidelines, institution of strong asthma care and education programs that are sensitive to local and individual patients’ and caregiver perceptions and experiences including emotional distress, the need to institute chronic care approach and ways to address patients’ medication access issues.

**Supplementary Information:**

The online version contains supplementary material available at 10.1186/s12890-022-01984-2.

## Background

Asthma is one of the common respiratory diseases affecting the communities globally with prevalence ranging from 10 to 13% [1]. In the African region, the prevalence of asthma especially in children less than 15 years increased from 12.1% in 1990 to 13.9% over two decades. In sub-Saharan countries, estimated prevalence varies widely between countries: Ethiopia 9.1%, Kenya 15.8%, Nigeria 13.0%, and South Africa 20.3% [2].

Asthma has been defined by the Global Initiative for Asthma as “a heterogeneous disease, usually characterized by chronic airway inflammation which is defined by the history of respiratory symptoms such as wheeze, shortness of breath, chest tightness and cough that vary over time and in intensity, together with variable expiratory airflow limitation” [3]. While asthma can develop at any stage in life, it most commonly develops in early childhood [4]. Its burden has been shown to excessively affect children and is said to be among the top ten chronic conditions for 5–14-year-olds. Asthma can be treated depending on the severity of the symptoms. According to the current Global Initiative for Asthma recommendations, the main goal of asthma treatment is to achieve and maintain good control of symptoms and inhaled corticosteroids (ICS) are recommended as one of the best maintenance therapies [3].

Non-adherence to asthma treatment is associated with poor asthma management, higher healthcare utilization, healthcare costs, and reductions in health-related quality of life [5]. In the management of childhood asthma, the issue of adherence is particularly essential and challenging because they are easily vulnerable to airway restructuring which can lead to permanent life-threatening airway obstruction [6]. The findings from studies in the west have depicted how factors related to the children’s and caregivers’ health beliefs about asthma and its treatment and their poor relationships with healthcare providers (HCPs) undermine effective asthma management [7]. Such studies could guide the development of interventions. They however need to consider local contexts including local cultures and thus the rationale for this study. The intent of this study was therefore to explore the perception of children with asthma, their caregivers and HCPs towards asthma and its management. The findings from this study which are expected to further knowledge on this issue in Ethiopia can guide policy makers and HCPs in addressing issues raised in the management of asthma in children.

## Methods

### Study design and settings

The study followed a descriptive qualitative approach [8] to enable an in-depth understanding of the perceptions of the children and their caregivers towards asthma and its management which has received limited attention to the best of our knowledge especially in the present context. The two sites selected for the present study were Tikur Anbessa Specialized Hospital (TASH) and St. Paul’s Hospital Millennium Medical College (SPHMMC), both public tertiary hospitals and located in Addis Ababa, Ethiopia. These hospitals run pulmonary follow-up clinics led by pediatric pulmonary and critical care physicians and serve the largest pool of children patients with asthma and thus the rationale for their selection as the study sites. The chest clinics normally give three to six months appointments to the children patients. These appointments however depend on the asthma control whereby those children with uncontrolled asthma may be given a one month or even less appointment date.

### Study participants’ selection

This study used purposive sampling technique to select children with asthma, their caregivers and HCPs including physicians, nurses and pharmacists as participants. This sampling technique was preferred to select study participants who could express their feeling independently and had more exposure with asthma and its barriers to treatment. The criteria used for children study participants and by their virtue for the caregivers were age of 8 to 15 years old, attending chest clinic follow-up for at least six months, using ICS for at least three months and ability to communicate either in Amharic or English and not being severely ill. Furthermore, effort was made to include a heterogenous sample by including study participants (children with asthma and their caregivers) who had different residence localities and severity levels of asthma to provide a diverse range of cases relevant to the topic. The criteria for the physicians and nurses was working with children for at least 3 months in the chest clinic while for the pharmacists it was working in the pediatric pharmacy during the study period.

The children and their caregivers were approached on their follow-up day, and given the information about the study. Upon their assent and consent respectively, they were appointed for interview. The selected children were paired with their caregivers but interviewed separately to capture their views without interference from their caregivers. Sampling and recruitment of participants had continued until saturation was achieved which was when “the major themes were identified and participants started expressing the same idea repeatedly” [9].

### Data collection

Data collection took place from April to June 2018 using semi structured interview guides which were adaptations from similar studies and also based on Kleinman’s explanatory model and Horne’s necessity—concerns model to enable exploration of patients’, caregivers’ and HCPs perceptions towards asthma illness and its treatment including adherence to recommended regimens and any possible discrepancies in view of the above concepts among the different actors. The interview guides attempted to capture the culturally determined process of making sense of asthma, ascribing meanings to symptoms of asthma, understanding causal attributions, and expressing suitable expectations of treatment and related outcomes [10–13]. The interview guide for the children and their caregivers included questions on their perceptions of asthma, its perceived cause as well as the sign and symptoms, use of asthma medications with focus on ICS, perceived necessity and concerns related with the medications, adherence to recommended regimens, perceived barriers to asthma management and relationship with HCPs (Additional file 1). The questions for the health care providers revolved around experiences with managing childhood asthma and medication-related counseling (Additional file 2).

The first author conducted the face-to-face interviews which ranged from 18 to 40 min at a convenient location such as the participant’s home, separate office from the clinic and cafeteria inside both hospitals but far from the pediatric chest clinic which allowed privacy for the participants. Furthermore, she approached the children in a friendly manner and gave especially the younger ones a piece of paper to draw or write something, and then started with very simple questions to ease their anxiety. All interviews were audio-recorded while observation field notes were taken to record the major points as well as the contextual and nonverbal conditions. Following the first few interviews, the collected data was transcribed and analyzed and discussions held with the second and last authors to guide further interviews. Furthermore, debriefings were carried out with these few participants to improve subsequent interviews.

### Data analysis

The audio-recorded data as well as field notes were transcribed verbatim and then translated into English. The first author and another researcher with experience in qualitative research methods independently coded the sampled data and then discussed the coding to develop coding frames for each of the interviews. In this regard, Kleinman’s explanatory model and the necessity-concerns model were used to define codes although active search was made to identify emerging codes. Then themes and subthemes were developed in the data based on the two models and other emerging ideas to describe patterns, relationships and trends between variables following the recommendations for descriptive qualitative analysis [8].

### Rigor and trustworthiness

The rigor of the findings was enhanced by different means. One is the use of different sources such as children with asthma, their caregivers as well as their HCPs which enabled rich and triangulated findings. On the other hand, the investigators were a clinical pharmacist, a pediatric pulmonary and critical care physician and a social pharmacist with experience in medicines use study and qualitative research methods which allowed the issue to be viewed from different perspectives. The first author who was also the data collector went through a process of reflexivity from the initial stages right through the data collection, analysis and report writing to enhance the rigor of the study. Furthermore, three HCPs and two caregivers’ study participants were given the chance to comment on their respective interview transcripts.

## Results

### Participants’ backgrounds

A total of 54 participants took part in the study of which 46 were children and their family caregivers while the rest were the HCPs caring for the children. Majority of the children with asthma (15/23) and their caregivers were recruited from TASH. Of the total child participants, majority were female (13/23), within the age range of 8 to 12 (19/23) and between the grades of 1 and 5 (14/23). With regards to the family caregiver participants, majority were Muslim, were married females, were in the age range of 35 to 45 and completed high school. It was also apparent that nine were patients with asthma as can be seen in Table 1. Among the eight HCPs, majority were females and physicians (Table 2).

**Table 1:** Socio demographic characteristics of caregivers

Characteristics	Number
**Sex**	
Female	15
Male	8
**Marital status**	
Divorced	4
Married	18
Widowed	1
**Age group**	
25 – 34	8
35 – 44	12
45 – 50	3
**Religion**	
Muslims	14
Christian	9
**Level of education**	
Unable to read and write	6
High school	10
Higher education (first degree and above)	7
**Monthly income**	
< 1000	3
1000 – 3000	15
> 3000	5
**Asthma profile**	
Patient with asthma	9
Not diagnosed with asthma	14

**Table 2:** Profile of health care providers

Characteristics	No
**Profession**	
Physician	4
Nurse	2
Pharmacist	2
**Sex**	
Female	5
Male	3

### Themes

The key findings from this explorative study were clustered into three key themes: low biomedical knowledge towards asthma, burden of asthma and management of asthma. The third theme for its part had three sub-themes, namely adherence to the biomedical treatment, use of traditional home remedies and religious healing and interaction with healthcare providers.

### Low biomedical knowledge towards asthma

Both children and their caregivers reported that they were not well informed about the meaning of asthma by the HCPs. Majority of children with asthma have described signs and symptoms of asthma as difficulty of breathing, coughing and sweating. Caregivers who were themselves were patients with asthma also thought that genetic predisposition solely causes asthma as shown by the following quote.

“In my opinion the cause is hereditary because I am also asthmatic.” (Caregiver 8)

Some of the physicians also described that the children had limited knowledge about the disease and admitted that they didn’t adequately work on creating awareness about the disease to the children and their caregivers which affected their medication taking behaviors.

“Majority of children with asthma and caregivers do not know much about the disease and its behavior…So, they discontinue the medication as soon as they get a relief and they come back when symptoms aggravate.” (Physician 1)

All of the physicians also believed that majority of children with asthma as well as caregivers, had no clear knowledge about the cause of asthma as depicted by the following quote.

“Personally, I don’t think that children with asthma and their caregivers have a clear knowledge about the cause and aggravating factor of asthma. For example, some of them believed that asthma can be transmitted through coughing and inhalation like Tuberculosis.” (Physician 3)

### Burden of asthma

Almost all children with asthma expressed the psychological and physical burden of asthma on their daily life. A notable example relates to the children recounting how asthma affected play time at school and relationships with school friends. Furthermore, majority of the children explained how their daily activities such as playing outdoors or their choice of meals were scrutinized by their caregivers which may have its own impact on their physical and social activity.

“I usually feel its impact while I play with my friends. There were times when I stopped playing because I was afraid that I might suddenly face difficulty of breathing in the middle of the game.” (Child 20)

“At school, most of the children don’t want to play with me because they believe that I couldn’t play or run as good as they do.” (Child 10)

“Since my mother fears that my asthma will be aggravated, she always yells at me whenever I went to play outdoors with my friends. Frankly speaking, I hate to stay at home.” (Child 8)

The children also explained that asthma made them feel worried and nervous. The emotional burden extends to worries related to their inability to administer the medicine by themselves. On the other hand, majority of caregivers reported that asthma caused feeling of dependency on the children. They also described that it made them shy and limited their communication with people. Fear of exacerbation of asthma was another thing both the children and caregivers worried as depicted by the quote below.

“My life has become different since I was diagnosed with asthma. Emotionally, I feel anxious in my daily activity.” (Child 1)

“I am so worried about my child’s condition in case she suddenly experiences exacerbation of asthma symptoms.” (Caregiver 8)

### Management of asthma

Both biomedical and non-biomedical approaches were described in the process of managing asthma. By and large, the biomedical approach was the mainstay of treatment as would be expected in this hospital-based study although traditional healing was reportedly used as discussed below.

#### Adherence to the biomedical treatment

The major therapy in the pediatric chest clinic was inhaled steroids as explained by the HCPs. Different issues were however identified with regards to implementing the biomedical treatment both on the side of the care providers as well as the recipients. The major issue raised from the providers side were not achieving the goals of asthma treatment which were controlling acute exacerbation, improving patient’s quality of life and side effects. They reported high patient load in the hospitals and the inability of the physicians to correctly follow the recommendations of pediatric asthma management as reasons which are depicted by the following quotes.

“Most of the time, we don’t clearly classify asthma as mild, moderate, mild persistent etc. Therefore, the treatment in our clinic is more of symptomatic. In addition, as we treat children with steroid, we don’t strictly follow step-up, step-down principle.” (Physician 2)

“Personally, I am not fully confident when to prescribe steroids for children since the diagnosis is not clear and it is too difficult once it is started. I may give them high dose instead of medium dose and not sure when to discontinue the drug.” (Physician 4)

On the care recipient side, majority of children and caregiver participants described how they didn’t take their medication regularly as recommended and instead discontinued if they had no problem of breathing. This may have led among other things to the use of an opened inhaler after three or four months.

“I am taking the ‘oxygen’ (ICS) whenever my symptoms aggravate, like when I have difficulty of breathing.” (Child 3)

Majority of children and their caregivers believed that their asthma is under control while using ICS which they describe as ‘oxygen’. Both participant groups appreciated the necessity of the ICS although its use was in the form of a reliever rather than as a controller medicine. There were some children however who revealed taking the prescribed medication under the coercive force of their parents.

“I think it is a better medicine because it will enable me to breathe when I get difficulty of breathing. I mean it gives me oxygen.” (Child 8)

“Mostly, I am forced to take the “oxygen” (ICS)… I wouldn’t take it if my father did not strictly follow my medication.” (Child 11)

The health care providers likewise explained as to how children with asthma were taking ICS for some time and would then discontinue as soon as they felt better. Some of the HCPs also reported that children considered ICS as a reliever medicine instead of the preventer medicine as it was intended, as can be seen in the following quote.

“Most of children with asthma were not taking ICS as we prescribed; they took it on a PRN (on need) basis by themselves.” (Physician 2)

The children and caregiver participants reported different concerns about the medicine that are related to its ‘bad’ taste and smell, and loss of smell and taste. In addition, they also reported difficulty of administration, fear of side effects and general dislikes that contributed to their decisions not to take it as illustrated by the following quotes.

“I don’t like its smell. In addition, my tongue will not sense anything for some time after I administer the ICS.” (Child 3)

“As I heard from my friends, ICS will cause some side effects like hypertension and I think it will cause my child to be dependent only on this medication.” (Caregiver 22)

Some caregivers also described that they faced some challenges from the society about their children’s asthma therapy as depicted by the following quote from one of the caregivers.

“My neighbors and even my wife tried to persuade me that I shouldn’t administer ICS to my child as they thought it will make her dependent on this medication. However, this didn’t make me change my mind…” (Caregiver 9)

All of the physicians believed that children with asthma did not take their prescribed medicines, especially the ICS, as recommended. They explained that the reasons may be negative perception about the medicine, fear of side effects and ‘creating adaptation’ and difficulty of administration.

“I think that majority of our patients are undertreated… usually they hesitate to begin steroid treatment but once they started, they will rush to discontinuing the steroid.” (Physician 3)

“The main concern is that majority of the caregivers had a bad perception about the ICS… For example, they thought the medicine will make adaptation and through time the patient will not respond to this medicine…” (Physician 2)

Furthermore, all participants including the health care providers complained about the unavailability of ICS and other essential medicines in the hospitals which would then force the caregivers to buy them at a private pharmacy at a much more expensive price. It was also cited by some participants that the medicines may sometimes not be available even in the private pharmacies. This was depicted below by the quotes from an emotional mother and her child as well as an HCP.

“I was so disappointed when I couldn’t find the medication when my child was very sick. I thought the hospital has a separate chest clinic unit and serves many children with asthma daily. So, it was hard to believe that this huge hospital lacked this medication.” (Caregiver 21)

“The ‘oxygen’ which was prescribed for me is not available in the hospital. For example, last time we couldn’t find the medicine. Then my mom was forced to buy it from private pharmacies. Currently, it is also not available everywhere (in town).” (Child 21)

“I don’t think that ICS as well as salbutamol is available in adequate amount in this hospital, I mean it (availability) is not constant.” (Physician 3)

#### Use of traditional home remedies and religious healing

Majority of the children with asthma as well as their caregivers cited that they used traditional home remedies as well as religious healing practices on a complementary and alternative basis. With regards to the home remedies, the most commonly mentioned were “*tazma mar*” (a special type of honey obtained from stingless bees), and other remedies that they believed will help with their asthma such as ‘milk with honey and garlic’. Caregivers also shared the use of preventive measures such as making adaptation at home and dietary modifications such as avoiding cold foods (e.g. ice-cream) and giving warm foods and drinks. Illustrations of the above can be seen in the following quotes.

“Sometimes, I will give him a special honey called “tazma mar” because, I heard that it is good for relieving cough and other symptoms of asthma.” (Caregiver 3)

“Oftentimes, I discontinued administering the medication and started natural treatment like dressing him in a sweater, giving him honey and milk with garlic.” (Caregiver 12)

There was also the practice of religious healing to help manage the asthma as can be seen in the quote below.

“We usually went to church to pray and I will drink holy water when my symptoms get worse.” (Child 3)

#### Interaction with healthcare providers

The interaction that the children and caregivers had with the HCPs can be described in generally as good and friendly. The high patient load in these tertiary hospitals however preclude the provision of adequate education about asthma. Some of the HCPs also expressed expectations that the children may have some knowledge about their condition given by the other colleagues which further reduced the amount of information provided. These ideas are depicted by the following quotes.

“I have a good relationship with the healthcare providers. I like their friendly relation with my child and he also liked them. So, he is happy when there is an appointment to this clinic.” (Caregiver 9)

“I think the children may not have adequate knowledge about the disease because we are not working on creating awareness about the disease due to patient load in the clinic.” (Physician 2)

“Since I believe that the doctors will advise them before coming to the pharmacy, I don’t usually advice about the disease. However, I will tell them little about the administration of ICS.” (Pharmacist 1)

## Discussion

The study revealed about the low biomedical knowledge of the child participants and their caregivers with regards to asthma and its management. Furthermore, both the children and their caregivers reported experiences of emotional, physical and social burdens related to asthma in their daily activities. Findings from both the HCPs, the children with asthma and their caregivers revealed issues in the management of asthma. The major issues reported from the HCPs side relate to the inability of the physicians to follow the pediatric asthma management guidelines. On the part of the children and their caregivers, there were issues with not taking the prescribed medications regularly citing different concerns including about their safety and access. Furthermore, the children and their caregivers revealed about their use of traditional home remedies and religious healing on a complementary and alternative basis. These issues may have led among other things to the reported suboptimal care and adherence problems which in turn led to symptom aggravation.

Children with asthma and to a certain extent their caregivers had reported emotional, physical and social burdens that have negatively affected their daily life and possibly different aspects of their health. Similar findings were reported by a study done in the UK among children aged nine to 16 years old where they reported social and emotional burdens such as feelings of embarrassments in taking medications among their peers and parents’ interference with peer interactions among other things [7]. Other studies reported from the United Kingdom [14], Hong Kong [15] and Taiwan [16] involving primary caregivers also reported different difficulties faced by these participants including fear and worry about the condition of their children, loss of confidence, feeling of powerlessness due to repeated hospital admissions of their children, feelings of uncertainty, feelings of chaos and instability, social tension and family conflicts. The findings of this study are to our knowledge the first from Ethiopia that describe such burdens among children with asthma and their primary caregivers. Such phenomena are bound to negatively affect the adherence to recommended treatment regimens and the health outcomes [17] and the well-being of both the children and their caregivers. This at the very least calls for further in-depth research on the experiences of these groups of participants including its impact on their health-related well-being so as to come up with appropriate policy and practice recommendations.

It was also apparent from the study findings that the management of childhood asthma was suboptimal and unsatisfying even for the HCPs. The issues mainly relate to the non-adherence of the physicians to follow the pediatric asthma management guidelines which seems to be common even in the western settings according to a review article [18]. Among the issues cited by this review article include high ICS prescription rates without diagnosis of persistent asthma or without formal diagnosis of asthma for that matter and prescription of ICS as short courses for symptomatic episodes. Also in relation to non-adherence to asthma treatment guidelines [3], this review reported issues with application of patient-centered communication skills [18] which may likewise have contributed to the suboptimal management of asthma reported by the present study despite the friendly approach followed by the HCPs.

As evidence to the suboptimal management, all groups of participants have described adherence issues among the child patients in implementing recommended treatment regimens related to the ICS, including its use as a reliever medication. This may have to do with limited awareness about its necessity but also concerns including about its side effects, ‘creating adaptation’ and loss of taste and smell. Similar observations are reported in studies done among Whites as well as Asian communities with the concerns and adherence issues more pronounced among the latter [7, 14, 15]. This variation could be partly attributed to cultural differences among the different communities which in turn could affect the trust towards the biomedical regimens. This calls for HCPs to follow patient-centered approaches as recommended by the asthma guidelines which includes due consideration for social and cultural contexts of the child patients and their caregivers during the provision of asthma education sessions and involving them in decision-making processes [3]. Involving some of these caregivers as peer asthma educators can also potentially improve asthma care and outcome [19] in the study setting where there is high social interaction.

On the other hand, better adherence to treatment and improved asthma control were reported by the review article when the care was provided by a specialist-led team that included allied health professionals [18]. In fact, such an approach that involved trained nurses running an asthma clinic was reported to be satisfying by primary caregivers to children with asthma in a study that involved White British and South Asian participants. These participants reported how they were able to access a regular asthma review session which also included additional support on inhaler techniques and peak flow meter use [14]. Experiences with nurse-led care are also reported in the resource limited settings of sub-Saharan Africa where an intervention study involving a nurse-led management program for asthma with the close support of physicians in Cameroon led to marked improvement in patient outcomes and was well received by the community [20]. While there are no reported studies of a similar experience in Ethiopia, the experience with HIV care and treatment can be leveraged to improve patient care for NCDs such as pediatric asthma through the chronic care model [21]. In the tertiary settings of the present study, nurse or pharmacist educators can be assigned following these models to provide requisite chronic care for these patients as part of the chest clinic. These providers can also be trained to assess for psychosocial issues so as to refer to appropriate section in the hospital.

Home remedies and religious healing were reportedly used on a complementary and alternative treatment basis in the majority of the children with asthma as would be expected in the Ethiopian setting especially for chronic conditions [22]. A study done among patients with asthma in south Ethiopia has in fact mentioned that the majority use traditional and religious healing methods as alternatives to their treatment which could contribute to their non-adherence to the biomedically recommended regimens [23]. The use of such treatment for asthma on alternative basis to the biomedical treatment is also reported in different settings especially in the eastern world where such medicines are culturally more acceptable on the one hand and as safer alternatives to the “chemical substances” [24, 25].

Such findings are further calls for HCPs to forge nonjudgmental discussions with their patients about the pros and cons of using these complementary and alternative treatments which in turn also hint at the need for HCPs to get updated information on such commonly used treatments. On the other hand, the commonly mentioned home remedies, especially “*tazma mar*”, is also mentioned by different studies from Ethiopia [26, 27]. These and evidences from other countries indicate the need to further investigate its role in the treatment of asthma [28].

Another major concern had to do with access to asthma medications especially the low availability of the ICS in both hospitals. Irrespective of the diverse manner of use of this group of medicines, the unavailability in these public hospitals could have detrimental impacts on the children’s life. The low availability of ICS is supported by a wider study that assessed the availability, pricing and affordability of beclomethasone, budesonide and salbutamol in 52 selected low- and middle-income countries including Ethiopia. The findings of this study revealed the low availability of these medicines with the figure for beclomethasone standing at 19% in public hospitals and a bit higher at 42% in private outlets. Nevertheless, these medicines were largely unaffordable to the majority of the population in a low income setting such as Ethiopia [29]. This calls for yet another policy intervention to improve the supply and financing of these medicines in the public sector and the community at large. The full implementation of the community and social health insurance scheme would be a good step in this regard [30].

### Strength and limitation of study

The study is the first in its kind in the country as well as in its triangulation technique which incorporated the data sets including children with asthma, caregivers and HCPs view point. However, the transferability of the findings may be limited to tertiary settings as the contexts for patients at primary health care tiers and private setups may be different. The present study was done in hospital setups where patients or care givers are to a certain extent informed about asthma during their follow-up. So, in-depth interviews in non-healthcare settings can additionally help to understand experiences with asthma including its management in the community. Individual interviews were used for this study which allowed in-depth exploration of individual perceptions and experiences and discussion into sensitive topics. The use of focus group discussion on the other hand may have allowed for more shared data although it may still have inhibited dissenting opinions. Finally, the authors have been conscious and reflected on their biomedical backgrounds at different stages of the research process to minimize but not eliminate such bias during the study design, implementation and interpretations.

## Conclusions

Children with asthma and their caregivers expressed low biomedical knowledge towards asthma and its management and reported facing different burdens. It was also apparent from the study findings that all participants, i.e., HCPs, the child patients and caregivers contributed to the suboptimal management of the children’s asthma because of issues related to but not limited to inability to follow the pediatric asthma management guidelines by the HCPs, concerns related with ICS such as difficulty of administration, fear of side effect and general negative attitude towards it and low availability and affordability of the ICS. This in turn may contribute to suboptimal health outcomes.

Therefore, there is a need to ensure the implementation of asthma guidelines which in turn may involve continuing training of the HCPs on the technical aspects of asthma treatment as well as patient-centered approaches, increase the awareness of the patients and their caregivers towards asthma and the treatment including ICS and work on its adherence in children in concordance with care givers and HCPs. This may call for the development of a chronic care model that can address not only a culturally adapted asthma education but also provide or facilitate psychosocial support for both the children with asthma and their caregivers. The medicines access issue is another issue that needs to be addressed including through hastening the community and social health insurance programs that are under different stages of implementation.

## Supplementary Information

**Additional file 1**. Interview guide for patients and caregivers.

**Additional file 2**. Interview guide for the healthcare providers.

## Data Availability

The datasets used and/or analyzed during the current study are available from the corresponding author on reasonable request.

## Abbreviations

HCPs: Healthcare providers
ICS: Inhaled corticosteroids
SPHMMC: Saint Paul’s Hospital Millennium Medical College
TASH: Tikur Anbessa Specialized Hospital
