# Supplementary material for: Perceptions towards childhood asthma and barriers to its management among patients, caregivers and healthcare providers: a qualitative study from Ethiopia
Source: BMC Pulm Med. 2022 May 8;22:184. doi: 10.1186/s12890-022-01984-2 (PMC9080199; doi:10.1186/s12890-022-01984-2)
Supplement: Supplementary file 1 — Additional file 1. Interview guide for patients and caregivers. [file 12890_2022_1984_MOESM1_ESM.docx]

**English version of interview guide for patient and caregiver of the study entitled, “**Perceptions towards childhood asthma and barriers to its management among patients, caregivers and healthcare providers: a qualitative study from Ethiopia”.

A. **Introduction/Ice breaker (Patient and care giver)**

1. How did you learn that you (your child) were asthmatic? Can you describe your (child’s) health? Can you describe signs and symptoms of asthma?

2. When did the asthma start? What do you think of the factors that caused your asthma? What do you think triggers your asthma?

3. Who follows your (the child’s) asthma? How did the physician/s and/or healthcare professionals describe asthma to you?

**Topic Guide: Children**

**Knowledge about Asthma and treatment**

1. How long have you had asthma?

2. How does your asthma affect you/make you feel? Prompt for: Physical and mental effects. How severe is your sickness? What do you fear about your sickness?

3. How do you manage your asthma? Can you describe factors that will affect to manage your asthma? How do you feel about your relationship with your parents and health care providers?

4. What do you think of the kind of treatment you should receive? What type of medication/s are you taking for your asthma? How do you administer it? Do you think you are following the recommendations prescribed by your physician?

5. What do you think of Inhaled corticosteroids (“Oxygen”)? What is its benefit to your asthma? (Explore the perception of advantages and disadvantages associated to this type of prescription).

6. Most of asthmatic children have difficulty administering Inhaled corticosteroids(“Oxygen”). How do you administer it? Do you follow the recommendations prescribed by your physician?

7. Why do you take (or not) the Inhaled corticosteroids (“Oxygen”) in the way you have just described? What do you fear about using Inhaled corticosteroids?

(Explore different possible sources of influence, and among them, which one was the most important for the patient).

**B. Consideration of medical recommendations**

1. What do you do when your asthma worsens? Have you consider increasing the dose or frequency of the inhaled corticosteroids (“Oxygen”)?How frequently you will administer it?

2. Why do you react this way when your symptoms worsen? (Explore different sources of influence, and if there are many, see which one was the most important)

C. **Patient-physician relationship**

1. How would you describe your meetings with the physician/s and health care professionals that treat/s your asthma? Is there anything you want to add?

**Topic guide: Primary care giver**

1. What type of medication is the child taking for his/ her asthma?

2. How do you administer it? Do you follow the recommendations prescribed by your physician? Why do you administer (or not) inhaled corticosteroids (“Oxygen”) in the way you have just described?

3. What do you think of inhaled corticosteroids (“Oxygen”)? (Explore the perception of advantages and disadvantages associated to this type of prescription and if relevant, see what could help or prompt them to take it).

4. Why do you administer (or not) inhaled corticosteroids (“Oxygen”) in the way you have just described?

(Explore different possible sources of influence, and among them, which one was the most important for the patient).

**D. Consideration of medical recommendations**

1. What do you do when your child’s asthma worsens? Have you consider increasing the dose or frequency of the inhaled corticosteroids? How frequently you will administer it?

2. Why do you react this way when the child’s symptoms worsen?

(Explore different sources of influence, and if there are many, see which one was the most important)

E. **Patient-physician relationship**

1. How would you describe your meetings with the physician/s and health care professionals that treat/s your asthma? Is there anything you want to add?

2. In your opinion, what is your role in the treatment of asthma? What is your role?
