# Supplementary material for: Perceptions towards childhood asthma and barriers to its management among patients, caregivers and healthcare providers: a qualitative study from Ethiopia
Source: BMC Pulm Med. 2022 May 8;22:184. doi: 10.1186/s12890-022-01984-2 (PMC9080199; doi:10.1186/s12890-022-01984-2)
Supplement: Supplementary file 2 — Additional file 2. Interview guide for the healthcare providers. [file 12890_2022_1984_MOESM2_ESM.docx]

**English version of interview guide for healthcare providers of the study entitled, “**Perceptions towards childhood asthma and barriers to its management among patients, caregivers and healthcare providers: a qualitative study from Ethiopia”.

**Topic Guide: Health care provider**

**F. Background**

1. To start could you introduce yourself and your clinical role?

2. How long have you worked at this clinic / respiratory medicine?

3. How often do you see children with asthma?

**G. Consultations with children with asthma**

1. What do you see as the main aims of the treatment/management you provide? How do you manage childhood asthma?

2. What are you trying to achieve when managing childhood asthma? What are the factors that will affect your management of childhood asthma?

3. Do you think children with asthma will take the inhaled corticosteroids as prescribed by the physician? Why?

3. What care or advice do you normally give to parents?

4. We are interested in children aged 8-15 years. Is there anything we need to particularly think about, when thinking about management in this age group?
